# Traces of Unconscious Mental Processes in Introspective Reports and Physiological Responses

**DOI:** 10.1371/journal.pone.0124519

**Published:** 2015-04-13

**Authors:** Leonid Ivonin, Huang-Ming Chang, Marta Diaz, Andreu Catala, Wei Chen, Matthias Rauterberg

**Affiliations:** 1 Eindhoven University of Technology, Department of Industrial Design, Designed Intelligence Group, Den Dolech 2, 5612 AZ Eindhoven, The Netherlands; 2 Universitat Politècnica de Catalunya, CETpD, Rambla de l'Exposició 59–69, 08800 Vilanova i la Geltrú Barcelona, Spain; Utrecht University, NETHERLANDS

## Abstract

Unconscious mental processes have recently started gaining attention in a number of scientific disciplines. One of the theoretical frameworks for describing unconscious processes was introduced by Jung as a part of his model of the psyche. This framework uses the concept of archetypes that represent prototypical experiences associated with objects, people, and situations. Although the validity of Jungian model remains an open question, this framework is convenient from the practical point of view. Moreover, archetypes found numerous applications in the areas of psychology and marketing. Therefore, observation of both conscious and unconscious traces related to archetypal experiences seems to be an interesting research endeavor. In a study with 36 subjects, we examined the effects of experiencing conglomerations of unconscious emotions associated with various archetypes on the participants’ introspective reports and patterns of physiological activations. Our hypothesis for this experiment was that physiological data may predict archetypes more precisely than introspective reports due to the implicit nature of archetypal experiences. Introspective reports were collected using the Self-Assessment Manikin (SAM) technique. Physiological measures included cardiovascular, electrodermal, respiratory responses and skin temperature of the subjects. The subjects were stimulated to feel four archetypal experiences and four explicit emotions by means of film clips. The data related to the explicit emotions served as a reference in analysis of archetypal experiences. Our findings indicated that while prediction models trained on the collected physiological data could recognize the archetypal experiences with accuracy of 55 percent, similar models built based on the SAM data demonstrated performance of only 33 percent. Statistical tests enabled us to confirm that physiological observations are better suited for observation of implicit psychological constructs like archetypes than introspective reports.

## Introduction

Recently, research in psychology has been rapidly expanding in the area of implicit (or unconscious) information processing [1,2]. Despite being considered a taboo topic not so long time ago, unconscious mental processes that define and motivate behavior of people have gained substantial attention in multiple disciplines ranging from cognitive psychology [3] to human computer interaction [4] to neuroscience [5]. The study of unconscious processes is particularly relevant to the domain of consumption because the practical implications of such processes are easily apparent. According to Zaltman [6], “ninety-five percent of thinking takes place in our unconscious minds—that wonderful, if messy, stew of memories, emotions, thoughts, and other cognitive processes we are not aware of or that we cannot articulate”. Although this statement made with regard to consumers may sound slightly extreme, Nisbett and Wilson [7] documented that individuals indeed may not be very well aware of and not able to report on their mental processes. A good showcase of the recent advances in consumer psychology related to unconscious processing can be found in [8]. Besides the consumer domain, the importance of unconscious processes is emphasized in new forms and theories of communication. Traditionally, communication concepts have a preference towards consciousness but with introduction of novel forms of multimedia communication, such as Kansei Mediation [9], this tendency may change. Finally, as Saariluoma et al. [10] pointed out, design of human-computer interaction paradigms could require thinking beyond folk psychological approaches. This necessity, for instance, was acknowledged in a concept of an entertainment system [11] that utilizes an unconscious flow of information coming from users in order to generate emotionally rich feedback.

Although the phenomenon of unconscious processes (or the unconscious) is still to be understood by the scientific community, it was operationally defined by Bargh [3] “in terms of a lack of awareness of the influences or effects of a triggering stimulus and not of the triggering stimulus itself”. An important distinction between unconscious and subliminal processing is highlighted in this definition. According to this point of view, unconscious mental processing is not necessarily associated with responding to subliminal stimuli and runs continuously as a parallel background process in the psyche [12]. While there are different theoretical frameworks that constitute ways of understanding and describing unconscious processes, we decided to have a closer look at the model of the unconscious proposed by Jung [13]. The motivation behind our interest to this model is justified by practical considerations that will be discussed later and the circumstance that one of the important concepts of Jungian framework—archetypes—found applications in personality psychology [14], marketing [15–18], and consumer psychology [19].

According to Jung, the psyche includes three levels [20]: consciousness, the personal unconscious, and the collective unconscious. Consciousness is seen as the external level of the psyche consisting of those thoughts and emotions that are available for one’s conscious recollection. The personal unconscious represents a repository for all of an individual’s feelings, memories, knowledge, and thoughts that are not conscious at a given moment of time. They may be retrieved from the personal unconscious with a varying degree of difficulty that depends on how actively they are being repressed. The term ‘collective’ reflects the fact that this part of the unconscious is universal and has contents and modes of behavior that are similar in all individuals [13]. The collective unconscious does not develop individually but is inherited and accommodates innate behavior patterns for survival and reproduction. Jung described the content of the collective unconscious as archetypes or pre-existent forms. Archetypes “are seen as prototypical categories of objects, people, and situations that have been in existence across evolutionary time and across cultures” [20]. Some examples of archetypes are: anima (the female aspect of the male psyche), mother, sky father, and wise old man [21]. According to Jung, there is a distinction between archetypal representations and archetypes themselves. While the representation is simply what someone experiences when a concept of, for instance, sky father occurs in one’s mind, archetypes themselves are different [21]. Jung regarded archetypes as fundamentally unobservable configuration whose existence can be established empirically in a variety of forms [22]. For instance, the archetype of mother may manifest itself in infinitely many forms, and yet, the one common characteristic of the ‘mother-idea’ always remains intact [21]. When an archetype becomes activated and is experienced with associated feelings and thoughts, it will result in a complex within the personal unconscious [20]. Jung wrote that a complex within the personal unconscious is an independently organized conglomeration of emotions that are specific to an individual and are products of interactions among a number of archetypes [13,20].

As we pointed out earlier, although the validity of Jungian model remains an open question, the concept of archetypes clearly became prominent in many research areas. For this reason, a further inquiry on archetypes and associated unconscious processing seems appropriate. In this paper, we will refer to individuals’ unconscious mental experiences related to archetypes as *archetypal experiences*. Similarly to emotional experience, archetypal experience emerges as content-rich events at psychological level and is instantiated by physiological processes [23]. Archetypal experience is not a conscious state and represents a prototypic composition of implicit emotions that are universal across cultures and time. Recent research indicated that archetypal experiences could be powerful determinants of one’s behavior, emotion, and decisions [14,16,17,24–26]. Therefore, a technique for evaluation of people’s archetypal experiences is likely to be in demand among researchers. For instance, this technique could be utilized for testing individuals’ responses to early prototypes of products or media.

When an archetype becomes active in one’s psyche the individual experiences a complex of emotions specific to this archetype. For this reason, a possible approach for observation of archetypal experiences involves measuring emotional responses of individuals related to archetypes. The problem of measuring and uncovering emotional experiences of people is not new. There is a growing body of research in psychology that targets this question. Common approaches to unveiling individuals’ emotions include application of introspective reports [27] and usage of physiological observations [28]. Collection of introspective reports about affective experiences is commonly facilitated by the Self-Assessment Manikin (SAM) technique [29]. The SAM technique became frequently used by researchers in various domains [30–32] and could be considered as a reliable instrument. The second approach also seems interesting because it looks for an objective evaluation of an individual’s mental experiences and is able to bypass conscious awareness. Moreover, it has recently been demonstrated that physiological observations methods may have a potential to provide an inside about more sophisticated implicit concepts and, in particular, about archetypal experiences of people [33].

In this paper we are not setting out to solve the theoretical questions around the unconscious aspects of the psyche or the validity of Jungian model, rather to examine how well complex unconscious psychological constructs like archetypes can be observed from introspective reports and patterns of physiological activations. At the beginning of this study, we had no specific preference for giving emphasis to archetypes except the practical rationale. Archetypes were an interesting candidate to facilitate investigation of unconscious mental processes for the practical reasons that included availability of a conceptual framework that describes the unconscious, presence of established institutes with expertise in archetypal symbolism, and existence of the database containing graphical content illustrating various archetypes [34]. More information regarding the organization which provided expertise in archetypal symbolism and the database with archetypal content will be provided in this and the next sections.

This research question was addressed in our experiment where the relationship between archetypal experiences, introspective reports, and physiological signals of 36 individuals was studied. Our hypothesis for this experiment was that physiological data may provide a better reflection of archetypal experiences because, according to Jung [13], archetypes are implicit and not readily available for conscious recollection. The archetypal experiences were elicited with film clips that were developed in collaboration with The Archive for Research in Archetypal Symbolism (ARAS) [34], which is an organization that since the early 1930s has been collecting and annotating mythological, ritualistic, and symbolic images from all over the world and possesses a profound expertise in archetypes and their representations. Apart from the film clips for elicitation of archetypal experiences, we also introduced clips to induce several explicit emotions. This way, we could later compare our findings with the state of the art on the explicit emotion recognition. The film clips were organized with categories in such a way that every category corresponded to one of the archetypes or explicit emotions. During presentation of the film clips, physiological signals modulated by the autonomic nervous system (ANS) of the subjects were monitored. We preferred to focus on the signals related to the ANS and avoid measurement of activations of the central nervous system (CNS) due to practical considerations. The CNS measures often impose considerable limitations on the design of studies. For instance, functional magnetic resonance imaging (fMRI) requires participants to be placed in a scanner. Another common CNS measure is electroencephalography (EEG). While this is a more flexible approach than the usage of an fMRI scanner, the necessity of wearing obtrusive equipment on the scalp does not help subjects to feel natural and relaxed during interaction with products or media. In our study we monitored the following ANS signals: electrocardiogram (ECG), skin conductance, respiration, and skin temperature. After every film clip, the subjects were required to provide an introspective report about their feelings using the SAM ratings. Upon completion of the study we used the collected data and information about categories of the film clips for training of several classification models. Then, prediction performance of the models built using the introspective reports was compared with the models based on the physiological data. Furthermore, in the evaluation of performance we distinguished between models specific to the archetypal experiences and the explicit emotions.

## Materials and Methods

### Ethics Statement

Written consent was acquired from each participant prior to the experimental sessions. This was a non-clinical study without any harming procedure and all data were collected anonymously. Therefore, according to the Netherlands Code of Conduct for Scientific Practice (principle 1.2 on page 5), ethical approval was not sought for execution of this study.

### Experimental Design

#### Stimuli

An appropriate set of stimuli was required for the elicitation of the archetypal experiences in the experiment. According to Jung, symbolic representations of archetypes have been present across cultures for thousands of years. They were commonly used in artwork, myths, storytelling, and continue to be employed in modern mass media [14]. Therefore, the set of stimuli can be constructed by extracting powerful archetypal appearances from a rich variety of media sources.

However, a decision has to be made not just about which archetypes should be selected but also regarding the type of media to use. Past research in affect elicitation have utilized different media types for emotion induction in laboratory conditions, including images and sounds [35,36], music [37], and films [38]. Although the focus of that research was on affect elicitation, it highlighted the pros and cons of each media type, and we assumed that the media with a high affective impact is also likely to have a large impact on unconscious processes of people. For this reason, film clips were chosen as a media type for the induction of archetypal experiences. They are effective in capturing the attention of individuals and have a relatively high degree of ecological validity, meaning that their dynamic display resembles real life scenarios [39].

Four archetypes (anima, animus, hero, and shadow) were selected for this study. The number of archetypes was a compromise: there were more interesting archetypes to study, but an increase in subjects’ psychological states would make the classification more challenging. For this reason, only films depicting the most common archetypes [13] formed our pool of stimuli. The archetypes of anima, animus and shadow were chosen based on the work of Jung [22]. The archetype of a hero was selected according to the description of the hero’s journey provided by Joseph Campbell [40], who studied stories about heroes in myths, literature, and religion across cultures, places, and time.

Film clips that embody these four archetypes needed to be selected. Similar to the previous studies that employed films [39] we obtained our clips by extracting fragments from full-length commercial movies. However, our choices had to be evaluated and, if necessary, corrected by external experts in the area of archetypal research. Therefore, as it was mentioned earlier, we sought collaboration with ARAS [34]. Based on the fruitful cooperation with ARAS and their feedback, our set of archetypal stimuli was constructed from the clips, which were obtained from the movies specified in Table 1. Copies of the film clips cannot be shared due to the fact that they were extracted from commercial movies. However, all of the movies are freely available on the market and we will provide additional editing instructions to produce exactly the same clips upon request.

**Table 1:** Sources of the film clips.

Film clip	Movie	Start	End
Archetypal experiences			
Anima	American Beauty [78]	0:15:02	0:17:20
		0:19:03	0:20:04
		0:36:09	0:37:28
		0:43:39	0:44:11
Animus	Black Swan [79]	0:46:40	0:49:24
		1:17:22	1:18:22
		1:19:13	1:20:48
Hero	Braveheart [80]	0:36:11	0:37:00
		0:38:10	0:39:05
		0:39:22	0:41:43
		0:47:21	0:49:01
		0:49:58	0:50:50
Shadow	Fight Club [81]	0:51:07	0:51:27
		0:59:18	1:01:50
		1:47:41	1:49:53
Explicit emotions			
Active-pleasant	Mr. Bean [82]	0:02:37	0:03:57
		0:04:54	0:08:45
Active-unpleasant	The Silence of the Lambs [83]	1:39:37	1:44:42
Passive-pleasant	The Lion King [84]	0:15:30	0:18:13
		0:45:19	0:46:48
		0:47:51	0:48:52
Passive-unpleasant	Forrest Gump [85]	1:02:21	1:07:31

The film clips for each archetype and explicit emotion were extracted from the movies specified in the table. The clips consist of one or more fragments that were cut from the movies at the times specified in the two last columns. The time format is hours:minutes:seconds.

Apart from the archetypal stimuli, we also included in the study four film clips for elicitation of explicit emotions. The purpose of the stimuli for explicit emotions was to facilitate the comparison of psychophysiological responses to them and the archetypal films. Emotion is commonly represented with the circumplex model [41]. For this study, we chose four emotional states in such a way that they uniformly covered the two-dimensional affective space. They were located in each of the quadrants of the affective space. Based on their location, we named these emotional states as following: active-pleasant, active-unpleasant, passive-pleasant, and passive-unpleasant. In the process of selecting film clips for the explicit emotions we were guided by the previous studies in this area [38,42]. The final results of our selection are presented in Table 1.

#### Participants

Thirty-six healthy people were recruited for the experiment. Most of them were undergraduate or graduate students. Ten participants had to be excluded from the analysis due to technical problems with wireless physiological sensors and one participant was excluded because he did not comply with the experimental procedure. Thus, only data from 25 subjects, consisting of 12 women and 13 men, was used in this study. Of these, 11 participants were from Europe, 10 participants were from Asia, 3 participants were from Middle East and one participant was from South America. The average age for the women was 23.0 years (SD = 1.9) and for the men 25.4 years (SD = 4.5). Each subject signed an informed consent form and was financially compensated for participation in the laboratory session that took around 2 hours.

#### Apparatus

**Fig 1:**
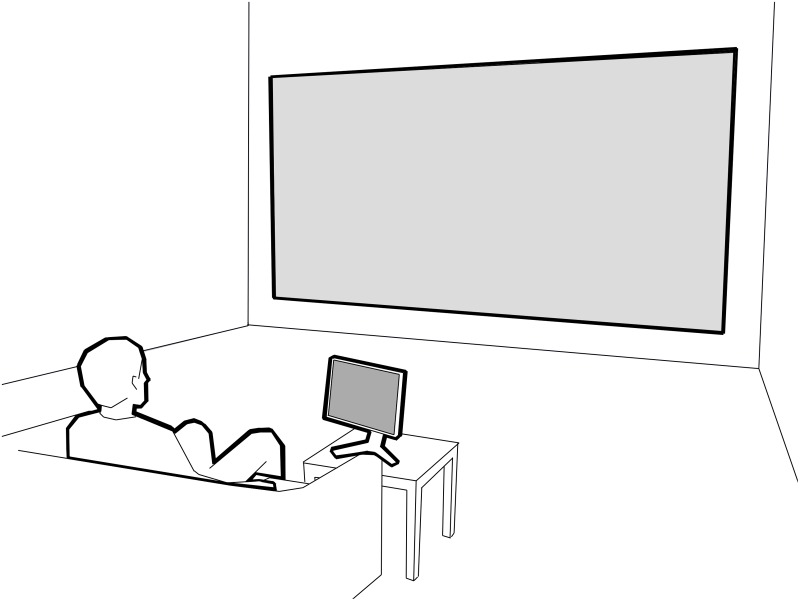
A subject is seated in the laboratory.

In a cinema like settings, film clips were projected on a white wall (592 x 222 cm) with a high definition beamer at a viewing distance of 4 meters. Additionally, a computer screen and a mouse were located near the couch where participants sat during the experimental sessions. After each film clip participants were asked to provide conscious feedback about their feelings by using the screen and the mouse. The setup of the experiment can be seen in Fig 1. Presentation of clips, collection of feedback, and time tracking were automated with a website developed for this experiment. Electrocardiography (ECG) and skin conductance of participants were monitored with Shimmer wearable wireless sensors [43] that streamed physiological data to a laptop via Bluetooth protocol. The three-lead Shimmer ECG sensor was connected with four disposable pregelled Ag/AgCl spot electrodes. Two of the electrodes were placed below the left and right collarbones and the other two were attached to the left and right sides of the belly. Similar electrodes were used to connect the Shimmer GSR sensor to thenar and hypothenar eminences of the participant’s palm on a non-dominant hand for measurement of the skin conductance. Unfortunately, due to the malfunctioning of the Shimmer ECG sensor, data for 10 participants had to be excluded from the analysis. For the measurement of the respiration and skin temperature, a Refa amplifier from TMSI BV was used in combination with an inductive respiration belt and a temperature sensor. The respiration belt of an appropriate size was strapped around the participant’s chest and the temperature sensor was attached to the subject’s belly.

#### Procedure

Upon registration for the experiment that took place several days in advance of an actual session subjects were asked to fill in a number of online personality questionnaires. One participant was studied during each session of the experiment. The session was started by inviting a participant to sit upright on the couch. The participant was asked to read and sign the provided informed consent form. Then, the experimenter explained how to place physiological sensors, helped the participant attach them, and made sure that the sensors streamed good quality signals. A time interval of approximately five minutes passed between placement of the sensors and presentation of the first clip. During this interval the electrode gel had enough time to soak into the skin, and thereby, ensure a stable electrical connection [44]. Meanwhile, the experimenter gave an overview of the study explaining that a number of film clips would be played, and the participant's physiological signals would be continuously monitored during the film's presentation. The actual goal of the experiment was not disclosed and, thus, the participant was unaware of the archetypes or emotions pictured in the clips.

**Fig 2:**
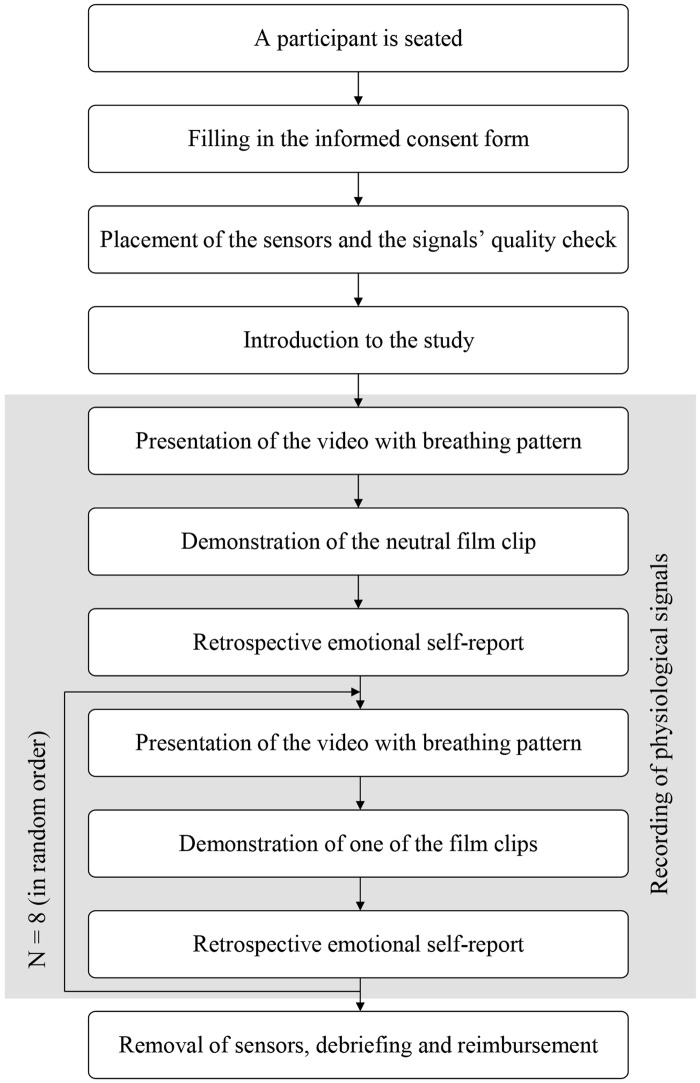
The procedure of the experiment is illustrated with a flowchart.

The participant was asked to find a comfortable sitting position and refrain from unnecessary movements. The light in the experimental room was turned off and the viewing experience was similar to the one in a movie theater. Since we wanted the participants to be in similar psychological and physiological conditions, demonstration of the films always started with a relaxing film. Piferi et al. [45] suggested using a relaxing aquatic video for establishing the baseline and provided experimental evidence that this is a better method for achieving the baseline than traditional resting. Next, films depicting the archetypal experiences and the explicit emotions were played in a random order. Before presentation of each film clip (including the first one), a short video demonstrating a breathing pattern was played. During this video that had duration of 40 seconds, the participant was asked to follow the breathing pattern (14 breaths per minute), and thereby, adjust their respiration rate to the common baseline. Immediately after each clip, the participant was asked to provide a retrospective emotional self-report using the computer screen and the mouse located near the participant’s dominant hand. The self-report data was collected with the SAM [29] that consisted of three sets of figures (valence, arousal and dominance). Every dimension of the SAM was characterized by a score from one to nine. When the participant completed viewing of the sequence of films, the light in the room was turned on and the sensors were detached from the participant’s body. Finally, the experimenter debriefed and reimbursed the participant. The procedure of the experiment is schematically presented in Fig 2.

#### Physiological Measures

Electrocardiogram (ECG) is a measurement of the heart's electrical activity over a period of time. It was recorded at 512 Hz and then treated with low-pass, high-pass, and notch filters. Filtering was necessary to remove high frequency noise (above 100 Hz), low frequency components, such as respiration (below 0.5 Hz), and mains hum (49–51 Hz). ECG is a rich signal, and it is commonly used in the psychophysiological domain for derivation of heart rate (HR) and heart rate variability (HRV). Heart rate is simply a measure of the number of heart beats per minute [46]. It was extracted from the ECG signal by detecting beats with an algorithm described in [47] and calculating the average heart rate over a non-overlapping moving window of 10 seconds. According to Kreibig [48], heart rate is the most often reported cardiovascular measure in psychophysiological studies of emotion. Thus, in this study we also expect to see a relation between the psychological states of individuals and their heart rate. Several HRV measures from time and frequency domains were calculated based on beat to beat intervals with HRVAS software package [49]. Time domain measures included the standard deviation of all beat to beat intervals (SDNN), the square root of the mean of the sum of the squares of differences between adjacent beat to beat intervals (RMSSD), and the standard deviation of differences between adjacent beat to beat intervals (SDSD) [50]. Frequency domain measures included total power, power in a very low frequency range (VLF, 0–0.04 Hz), power in a low frequency range (LF, 0.04–0.15 Hz), power in high frequency range (HF, 0.15–0.4 Hz), and ratio LF/HF [44]. HRV components have a long history of application in psychophysiological research [51] and have become important measures of individuals’ psychological states.

Skin conductance characterizes the electrodermal activity of skin and is related to changes in eccrine sweating, which are regulated by the sympathetic branch of the autonomic nervous system [44]. It has been proven that skin conductance is closely related to psychological processes and particularly to arousal [52]. Skin conductance consists of tonic and phasic components. The tonic component corresponds to relatively slow changes in skin conductance over longer time intervals, which can last from tens of seconds to tens of minutes. It is indicative of a general level of arousal, and thus, is called skin conductance level (SCL). On the other hand, the phasic component or skin conductance response (SCR) reflects high frequency variations of the conductivity and is directly related to observable stimuli [44]. Skin conductance signal was sampled at 256 Hz. In order to obtain SCL from the raw skin conductance signal a low pass filter was set to 1 Hz. For SCR, a high pass filter was additionally set at 0.5 Hz.

Respiration is another important physiological signal commonly employed in psychophysiological research [53]. It is related to changes in the sympathetic nervous system and can be used to determine psychological states of subjects [54]. The raw signal was filtered with low pass and high pass filters at 10 Hz and 0.1 Hz respectively in order to remove unnecessary noise. The respiration rate (RR) was calculated according to the recommendations provided by the manufacturer of respiration measurement hardware (TMSI BV). RR was then averaged over the duration of a film clip with a non-overlapping moving window of 10 seconds.

Skin temperature (ST) varies due to localized changes in the blood flow defined by vascular resistance or arterial blood pressure, which are consequently modulated by the sympathetic nervous system [55]. The variations in ST caused by confrontation of individuals with affective stimuli have been previously reported in the literature [56] and justify our decision to include ST into this study. ST is a slow changing signal, and thus, the raw data was harmlessly resampled to 64 Hz in order to speed up the calculation. A low pass filter of 10 Hz was applied to the resampled signal for removal of high frequency noise. Finally, ST was smoothed with a non-overlapping moving window of 10 seconds.

### Data Mining Techniques

#### Normalization

Physiological signals vary highly between subjects, so the primary goal of normalization is to reduce the variance and make physiological data from different individuals comparable. As this study involved more than one participant, the data had to be normalized. There are different approaches to the normalization of physiological data [57]. We chose to utilize the approach that involves subtraction of baseline values from the data corresponding to stimuli presentations. The result of the subtraction was then normalized to a range from 0 to 1 for each physiological signal separately using Eq 1 where *X*_*i*_ depicts a data point, *X*_*min*_ corresponds to the minimum value of a given signal, and *X*_*max*_ represents the maximum value of the signal. This normalization method was well suited for the design of our experiment where a baseline condition was recorded before each film clip.

$$X_{i}=\frac{X_{i}-X_{\min}}{X_{\max}-X_{\min}}$$

#### Extraction of Features

In this study, film clips with duration of approximately five minutes were shown to subjects, and thus, the physiological data that was recorded for each stimulus formed temporal sequences. Having reviewed the relevant literature, we identified several common types of sequence classification methods [58]:

- Feature based classification that involves the calculation of features that describe time series and then the application of them in conventional classification methods for static data.
- Sequence distance based classification. Frequently used distance functions include Euclidian distance and dynamic time warping distance (DTW).
- Model based classification. The Hidden Markov Model (HMM) is the most popular instrument for temporal classification and widely applied in the speech recognition domain.

We found the feature based method to be more suitable for a number of reasons. First, it provides a convenient way to include non-temporal attributes, such as some HRV features or gender of the subjects, into the analysis, which DTW and HMM do not [59]. Second, contrary to HMM, this method does not require a large amount of training data [59]. Third, the creation of template streams in the sequence distance method, which would represent a typical time series corresponding to psychological states, is not trivial.

In order to pursue classification, feature vectors had to be extracted from the collected data sequences. Taking into account the characteristics of our experiment, such as relatively short length of the stimuli, the approach to extraction of feature vectors with the technique known as segmentation was used [60]. This approach requires division of a time axis into several equal-length segments, and then, average values of temporal data along these segments are taken as attributes. The length of the segments was empirically chosen as 10 seconds, and therefore, each physiological recording provided 30 features per a film clip.

We proceeded with the application of this method to the physiological signals. A total of 38 features were obtained from ECG data: 30 features were extracted from HR temporal data based on the average value of HR calculated along non-overlapping segments of 10 seconds; eight conventional HRV features described earlier were taken without any modification (SDNN, RMSSD, SDSD, total power, VLF, LF, HF, and LF/HF). The skin conductance signal was compressed to a vector consisting of 60 features. First, 30 features were obtained from SCL, averaged over 30 segments of 10 seconds each. The remaining 30 features were extracted by averaging the absolute amplitude of SCR along the same 30 segments. Respiration data was reduced to 30 features that represented the average RR calculated for each of 30 segments. Finally, 30 features were obtained from the skin temperature signal and averaged in a similar manner over 30 segments. Therefore, in total 158 features were prepared for the classification.

#### Statistical Analysis

Although the primary goal of our experiment was to build and compare classification performance of the prediction models trained on the physiological data and the introspective reports, it was also beneficial to perform several statistical tests. These tests could help us to answer the question regarding significance of the relationship between the patterns of physiological signals, the SAM rankings provided by the subjects, and the labels of affective states induced with the film clips. Our study had a repeated-measures design because physiological measurements were taken from the same individuals under changing experimental conditions. An appropriate statistical test for this type of experiment would be linear mixed models (LMM). LMMs are parametrical statistical models for clustered, longitudinal or repeated-measures data that characterize the relationships between continuous dependent variables and predictor factors [61,62]. LMMs were used to investigate the statistical relationship between the physiological data and the labels of affective states. Additionally, we used multivariate analysis of variance (MANOVA) for repeated measures [63] as a test for analyzing the differences in the SAM rankings based on the categories of affective states induced with the film clips.

A software implementation of statistical procedures included in SPSS Version 19 (SPSS, Inc.) was utilized to answer the research questions pointed out earlier. Physiological responses of the subjects were treated as dependent variables (continuous responses), the film clips represented fixed variables and the physiological baselines measured during the presentation of the video with a breathing pattern before each stimulus were used as covariates. The LMMs interaction effect among categories of the film clips and physiological baselines recorded prior to the demonstration of the clips tests whether the patterns of the participants’ physiological responses are different between various stimuli. The SAM rankings were included in the MANOVA as dependent variables while the categories of the video clips served as independent variables.

Besides, paired samples t-tests were used for comparing classification accuracies of prediction models trained based on different data sets. All statistical tests used a 0.05 significance level.

#### Dimension Reduction

In data mining, it is important to identify an optimal number of features that are relevant for building an accurate prediction model. If number of the features is very large, the feature space volume also becomes vast, thus making the classification difficult. Moreover, if a training sample is not significantly large, overfitting may occur and lead to poor predictive performance. Therefore, it is beneficial to reduce the number of features as much as possible in order to build a computationally efficient and robust model for classification [64]. Various techniques have been proposed for feature reduction, including principal component analysis (PCA). This method has been applied in psychophysiological studies and enable the transformation of a large number of features into a smaller set of uncorrelated components [57]. Dimension reduction with PCA transformed 158 features into 25 components. Overall, we had 25 samples per each class. There were eight classes that corresponded to either one of the archetypes or one of the explicit emotions. The data of different subjects were combined in a single data set.

#### Selection of Classification Method

Once time sequences of physiological data were transformed into feature vectors, three types of classifiers were used in order to obtain a predictive model. These classifiers were chosen based on the history of their previous applications in recognition of emotions [57]. K-nearest neighbor (kNN) is a simple and transparent classification algorithm that works reasonably well with small samples of data and can be useful as a baseline measure for the judgment of performance achieved with other methods. The second selected classifier was naïve Bayes, which builds a probabilistic model based on training data and then assigns each feature vector to a particular class. The third classification method was linear discriminant analysis (LDA). This algorithm is transparent, meaning its results can easily be understood and interpreted by humans and it is easy to implement [57]. In order to ensure that a classification algorithm is not trained and tested on the same data set, a cross-validation technique was employed. We chose leave-one-out cross-validation because it provides an accurate assessment of the classification rate.

## Results

**Fig 3:**
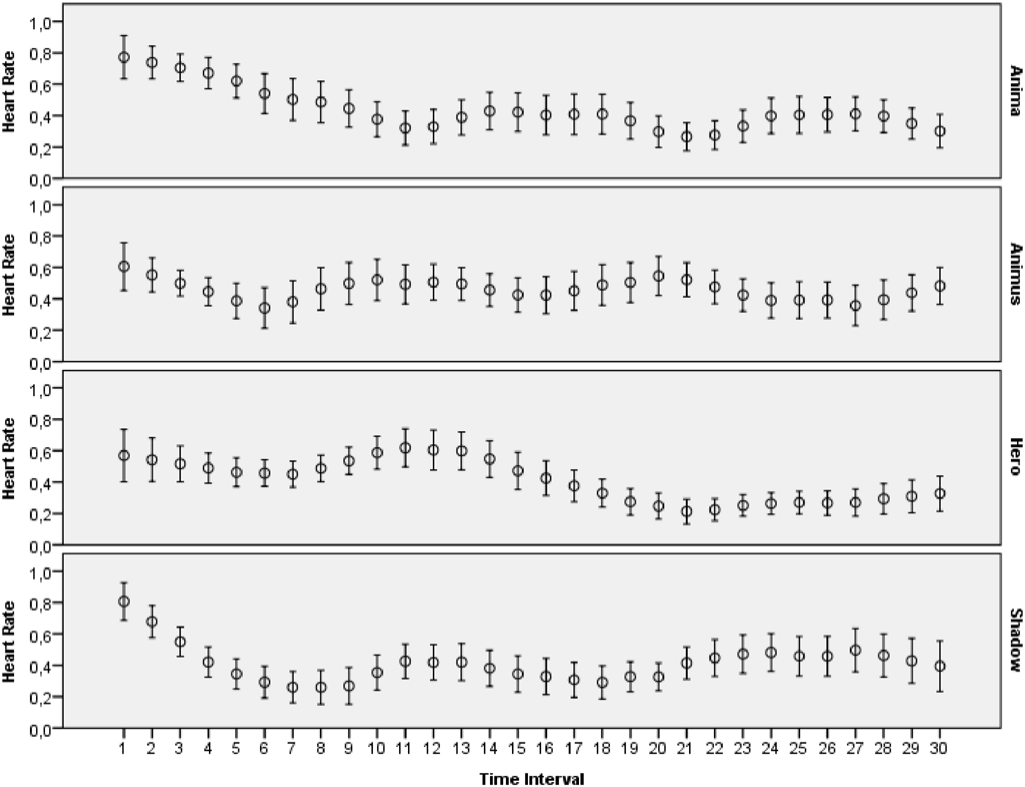
Mean values and 95% confidence intervals of heart rate signal related to the stimuli for archetypal experiences.

**Fig 4:**
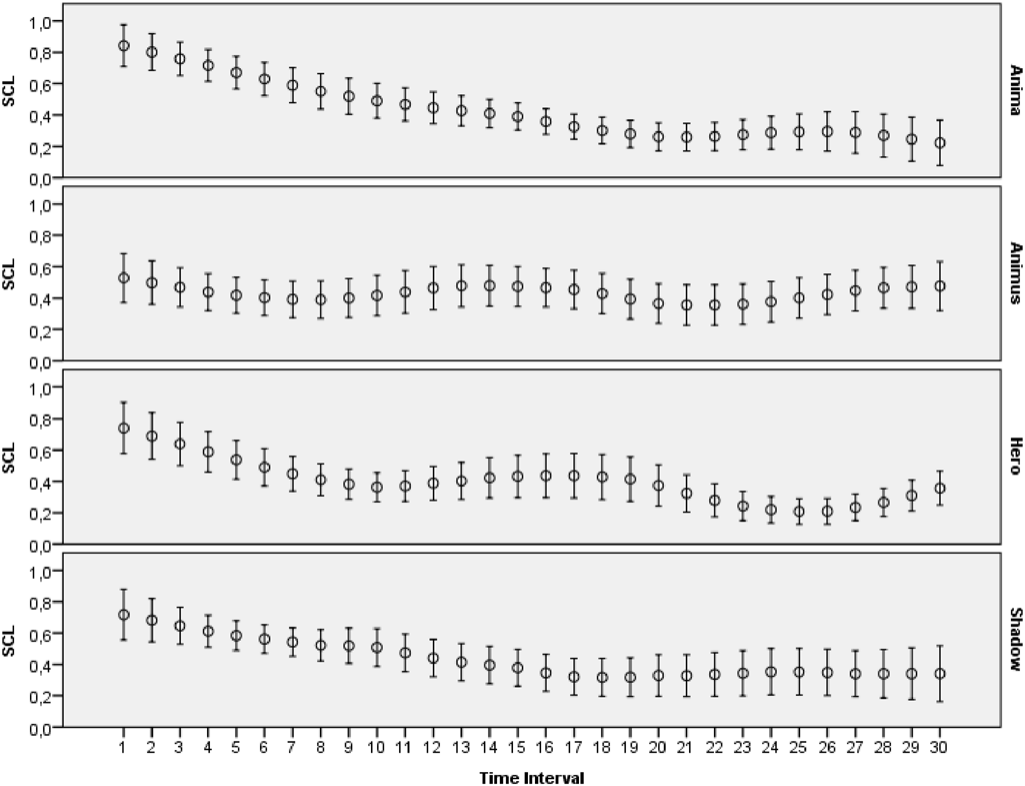
Mean values and 95% confidence intervals of skin conductance level (SCL) related to the stimuli for archetypal experiences.

**Fig 5:**
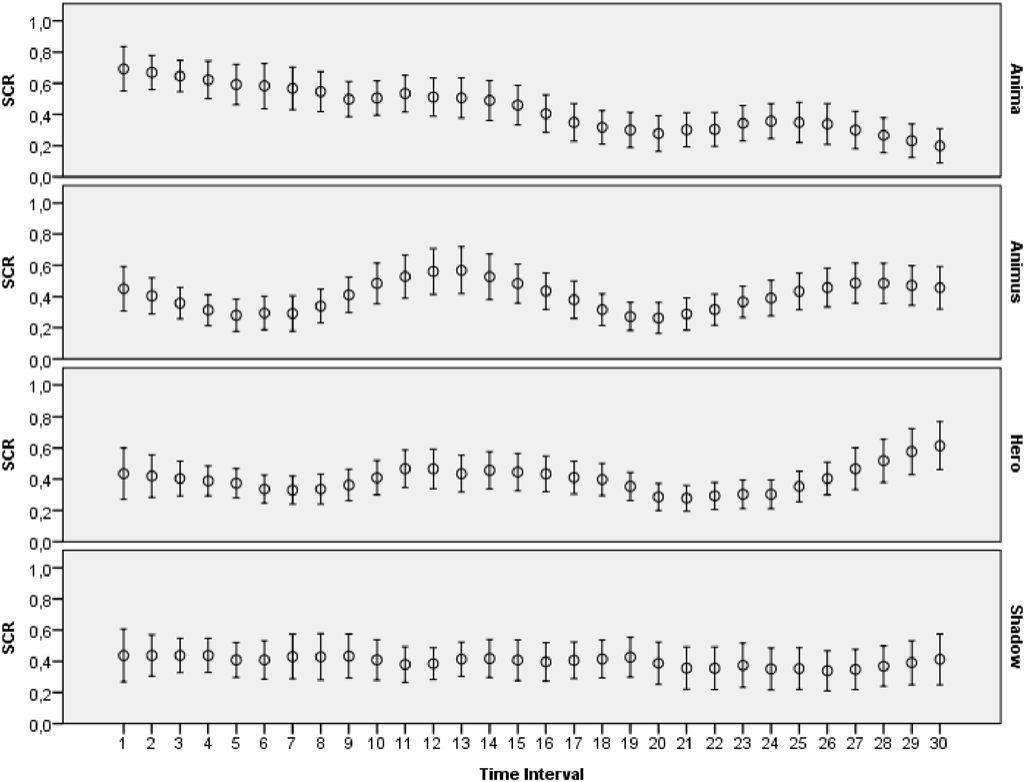
Mean values and 95% confidence intervals of skin conductance response (SCR) related to the stimuli for archetypal experiences.

**Fig 6:**
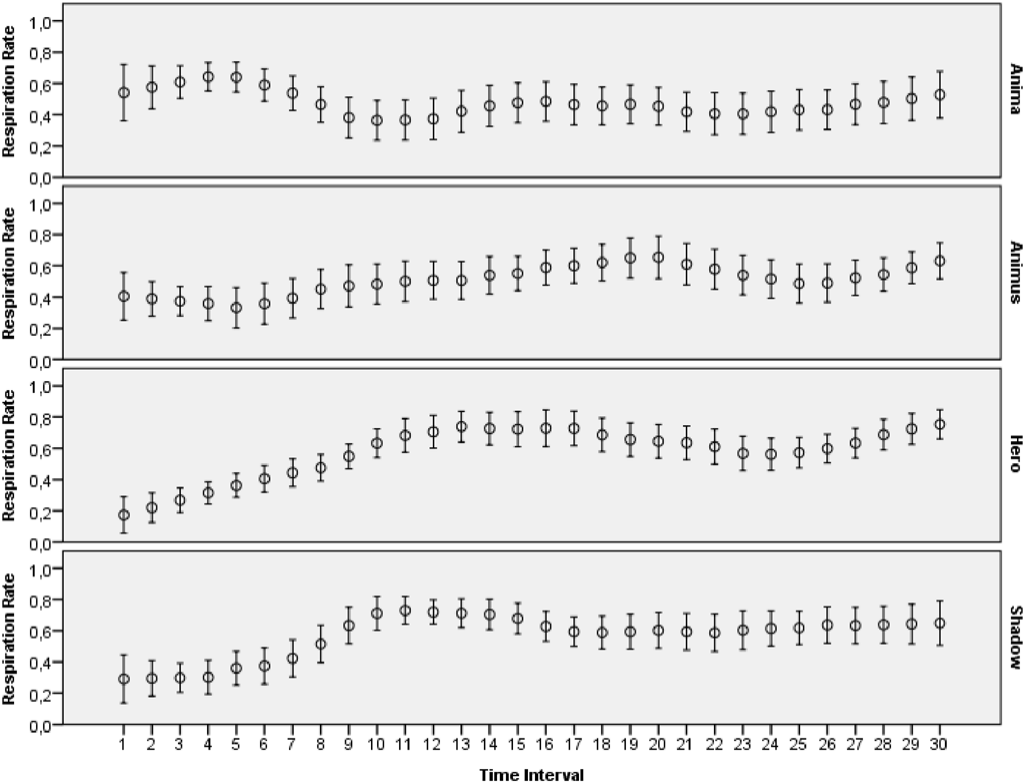
Mean values and 95% confidence intervals of respiration rate signal related to the stimuli for archetypal experiences.

**Fig 7:**
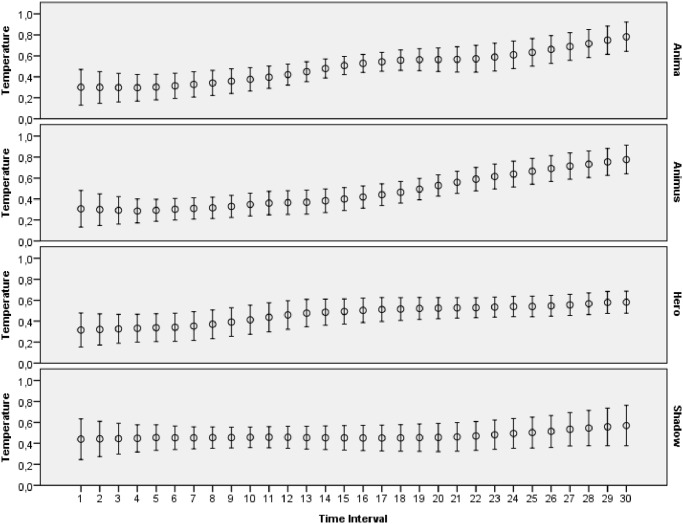
Mean values and 95% confidence intervals of skin temperature signal related to the stimuli for archetypal experiences.

**Fig 8:**
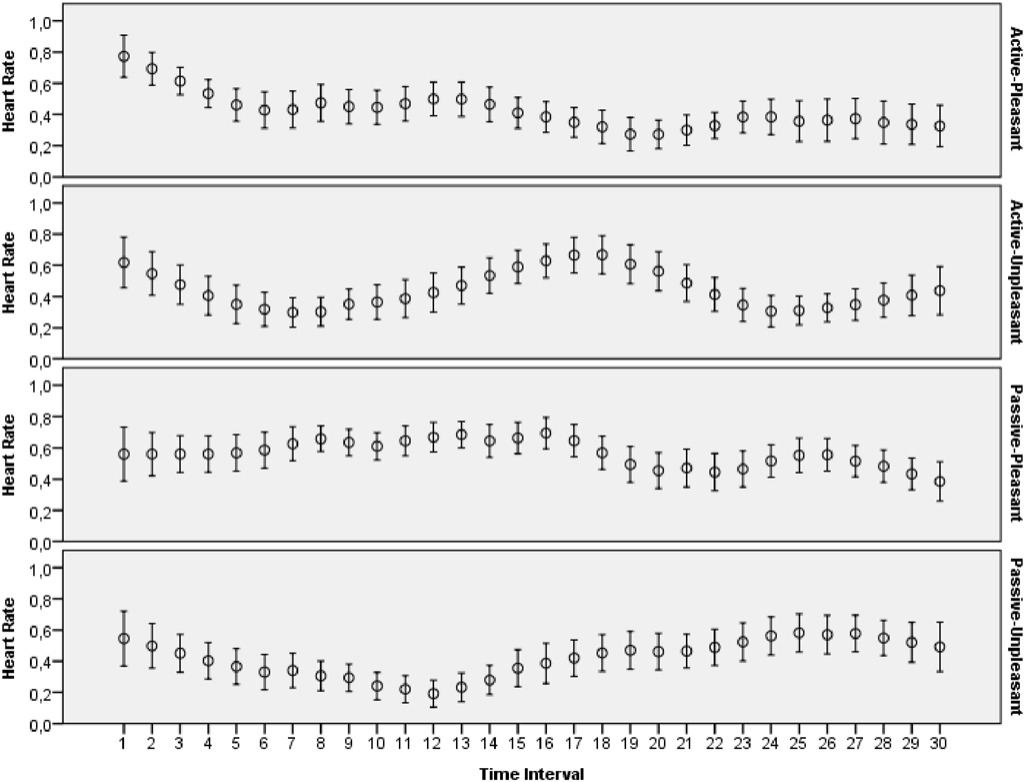
Mean values and 95% confidence intervals of heart rate signal related to the stimuli for explicit emotions.

**Fig 9:**
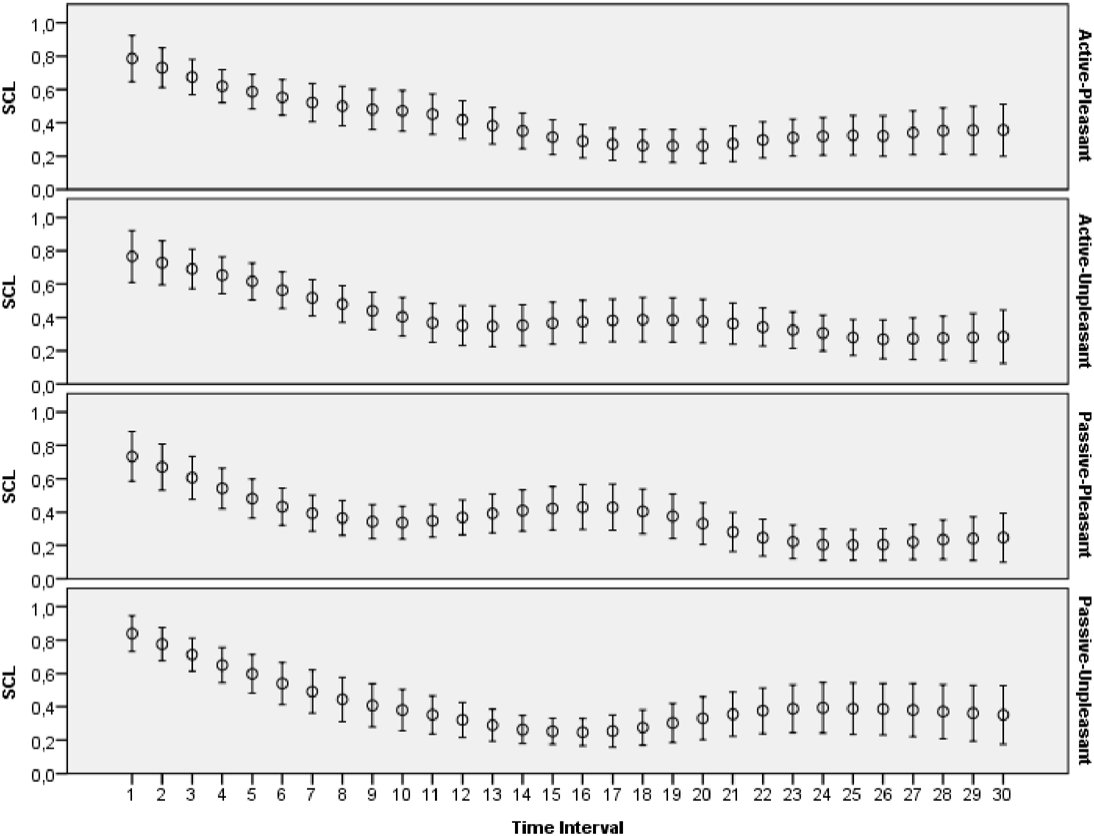
Mean values and 95% confidence intervals of skin conductance level related to the stimuli for explicit emotions.

**Fig 10:**
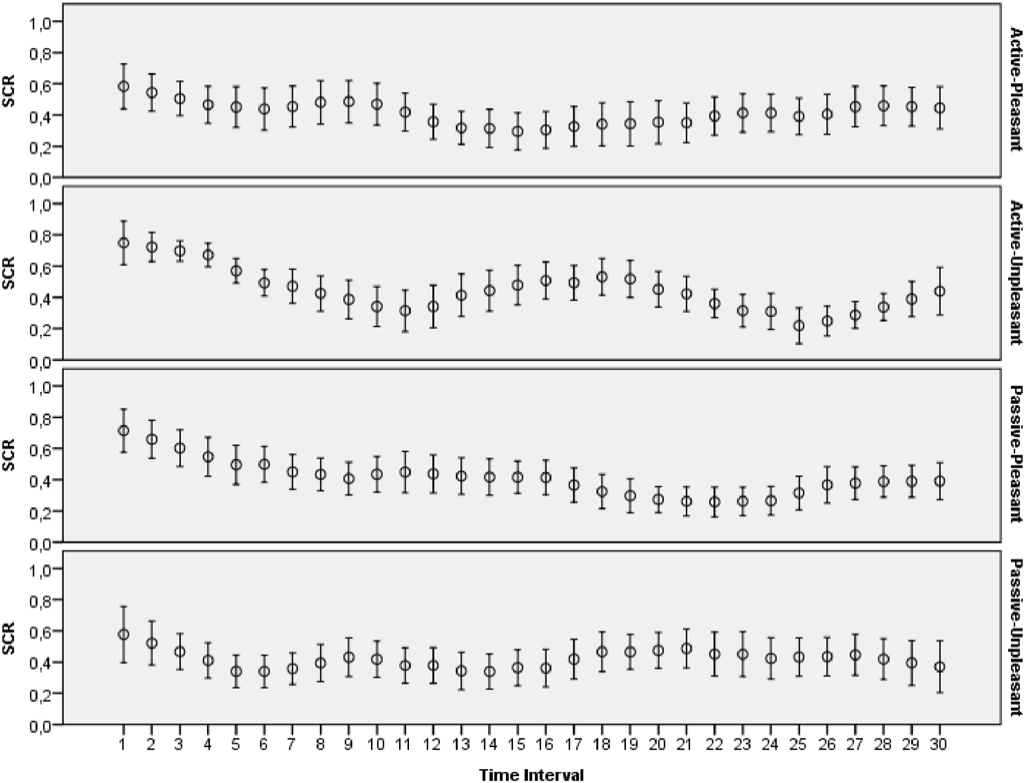
Mean values and 95% confidence intervals of skin conductance response related to the stimuli for explicit emotions.

**Fig 11:**
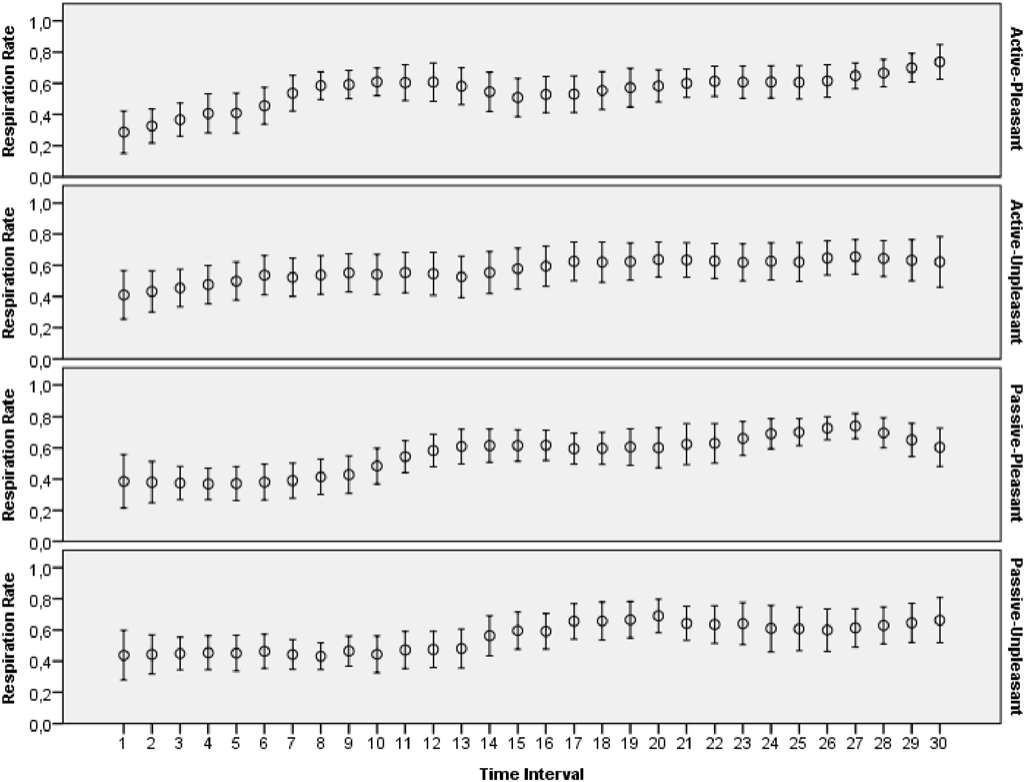
Mean values and 95% confidence intervals of respiration rate signal related to the stimuli for explicit emotions.

**Fig 12:**
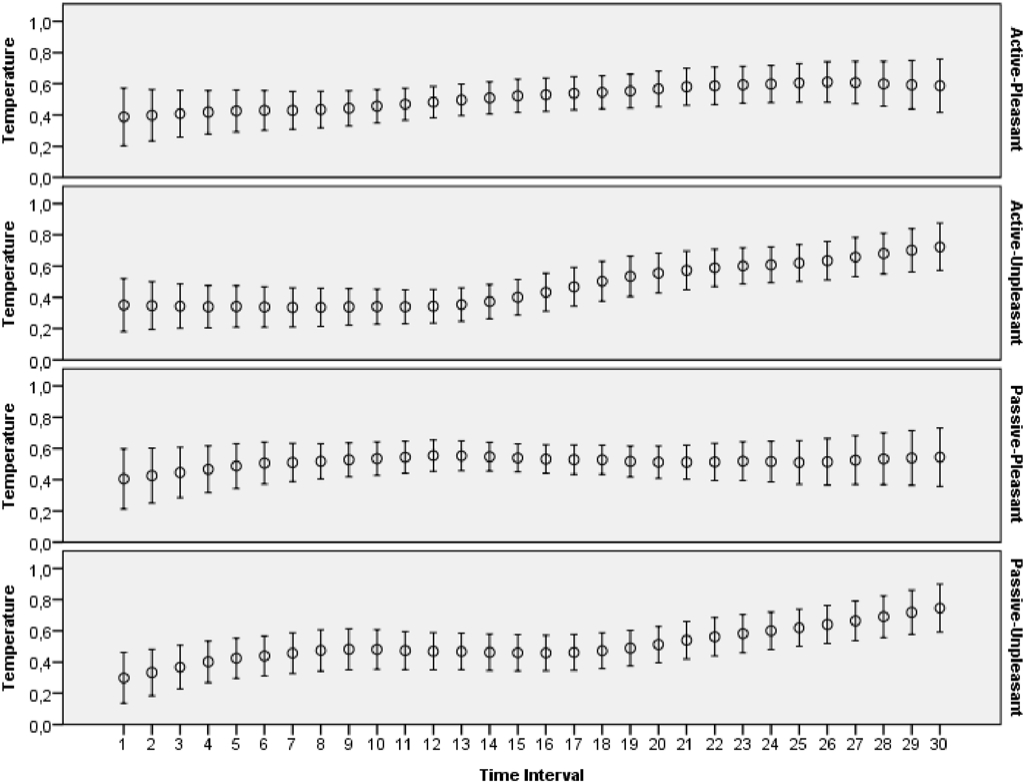
Mean values and 95% confidence intervals of temperature signal related to the stimuli for explicit emotions.

The main goal of this study was to evaluate how archetypal experiences of people are reflected in introspective reports and patterns of physiological activations. The features extracted from ECG, skin conductance, respiration and skin temperature recordings were arranged to form two data sets: one with the data for the explicit emotions, another with the data related to the archetypal experiences. All the data collected during this study was made publicly available online [65]. Moreover, several plots that demonstrate mean values and 95 percent confidence intervals of the physiological signals are presented on Figs 3–12. The mean values and confidence intervals were calculated using data of all the participants who watched a particular film clip. Descriptive statistics for the SAM ratings provided by the participants is presented in Tables 2 and 3 below.

**Table 2:** Descriptive statistics for the SAM ratings related to the stimuli for archetypal experiences.

	Anima			Animus			Hero			Shadow		
	Mean	SE	SD	Mean	SE	SD	Mean	SE	SD	Mean	SE	SD
Arousal	5.320	0.304	1.520	6.400	0.379	1.893	5.720	0.354	1.768	6.080	0.416	2.080
Valence	5.880	0.318	1.590	3.680	0.304	1.520	4.520	0.421	2.104	5.000	0.424	2.121
Control	5.560	0.404	2.022	4.360	0.424	2.119	6.040	0.438	2.189	5.08	0.458	2.290

The SAM ratings were measured on the scale from 1 to 9. SE: Standard Error; SD: Standard Deviation.

**Table 3:** Descriptive statistics for the SAM ratings related to the stimuli for explicit emotions.

	Active-Pleasant			Active-Unpleasant			Passive-Pleasant			Passive-Unpleasant		
	Mean	SE	SD	Mean	SE	SD	Mean	SE	SD	Mean	SE	SD
Arousal	4.160	0.502	2.511	6.600	0.473	2.363	4.360	0.538	2.691	4.120	0.514	2.571
Valence	7.400	0.294	1.472	3.040	0.344	1.719	8.320	0.150	0.748	4.840	0.415	2.075
Control	6.640	0.351	1.753	3.160	0.489	2.444	7.200	0.332	1.658	4.640	0.428	2.139

The SAM ratings were measured on the scale from 1 to 9. SE: Standard Error; SD: Standard Deviation.

### Statistical Analysis

The statistical analysis was performed separately for each of the physiological signals monitored during the study. The primary goal of this analysis was to answer the question whether the mental experiences of the participants elicited with the film clips had a significant effect on the patterns of their physiological signals. First, LMMs were fit to each of the data sets with the HR features. The analysis, which the HR entered as a dependent variable, demonstrated a significant relation between the HR features and the interaction of the film clips’ categories and the HR baselines for both data sets: the explicit emotions data set, [F(3, 346.962) = 2.805, p = 0.040] and the archetypal experiences data set [F(3, 556.462) = 5.605, p = 0.001]. Next, we performed the same statistical tests for the SCL and SCR signals. The results indicated that there was a significant relation between the categories of the film clips and the SCL data points for the explicit emotions data set [F(3, 1263.444) = 14.634, p < 0.001] and the archetypal experiences data set [F(3, 1971.300) = 18.049, p < 0.001]. Similar findings were obtained for the SCR signal since the test demonstrated a significant relationship between the SCR baselines and the interaction among the category of film clips and the SCR baselines on the explicit emotions data set [F(3, 469.721) = 34.528, p < 0.001] and the archetypal data set [F(3, 660.484) = 29.718, p < 0.001]. Then, we fit LMMs for the RR signal that was collected. The statistical analysis indicated a significant interaction effect for the explicit emotions data set [F(3, 444.367) = 4.909, p = 0.002] but not for the archetypal data set [F(3, 531.813) = 2.305, p = 0.076]. Finally, we carried out analysis with LMMs for the skin temperature data. The results related to the relationship between the dependent variable (skin temperature) and the interaction among the film clips’ categories and the physiological baselines demonstrated statistical significance for both data sets: the explicit data set [F(3, 2035.610) = 19.997, p < 0.001] and the archetypal data set [F(3, 2941.863) = 14.992, p < 0.001].

Having completed the statistical analysis of physiological data we proceeded with performing statistical tests for the SAM rankings provided by the participants. First, a MANOVA test was applied to the part of the SAM rankings related to explicit emotions. It indicated a statistically significant difference in the SAM rankings based on the category of the film clips [F(9, 16) = 31.804, p < 0.001 (Wilks’ Lambda)]. Similar findings were obtained for the SAM rankings related to the archetypal experiences [F(9, 16) = 5.755, p = 0.001 (Wilks’ Lambda)].

### Classification

We started by training prediction models using three different classification algorithms based on physiological data prepared with the data mining techniques described earlier. Then, the analysis on each of the data sets was performed using the extracted features as attributes. The archetypes or the explicit emotions presented in the clips served as class labels.

First, the archetypal data set entered our analysis. The model constructed with the kNN method was able to correctly classify 46 percent of the left out instances. The best performance was achieved with k = 20 neighbors. The k parameter was chosen heuristically. The overall accuracy of the model obtained with the naïve Bayes classifier was 55 percent. Finally, the LDA classifier was employed in building a prediction model. The obtained prediction model featured a classification rate of 53 percent, which was close to the one achieved with the naïve Bayes method. The classification results corresponding to different classification algorithms are presented using confusion matrixes in Table 4, Table 5, and Table 6. Each column of the confusion matrixes represents instances of the predicted classes. On the other hand, each row of these matrixes represents instances of the actual classes.

**Table 4:** Confusion matrix of kNN classifier on the archetypal data set.

Classified as →	Anima	Animus	Hero	Shadow
Anima	17	3	5	0
Animus	7	5	10	3
Hero	6	0	18	1
Shadow	7	2	10	6

**Table 5:** Confusion matrix of Naïve Bayes classifier on the archetypal data set.

Classified as →	Anima	Animus	Hero	Shadow
Anima	14	4	3	4
Animus	1	13	6	5
Hero	1	5	15	4
Shadow	4	4	4	13

**Table 6:** Confusion matrix of LDA classifier on the archetypal data set.

Classified as →	Anima	Animus	Hero	Shadow
Anima	14	4	2	5
Animus	2	11	4	8
Hero	2	1	19	3
Shadow	3	6	7	9

Next, we analyzed the data related to the explicit emotions. Our results indicated that the kNN algorithm could differentiate the emotional states with an accuracy of 42 percent (k = 25). The naïve Bayes classifier was able to predict the emotional states presented in the film clips with an accuracy of 40 percent. Similarly, the prediction model obtained with the LDA classifier could properly classify 47 percent of the instances. The outcome of the analysis is presented in Table 7, Table 8, and Table 9 using confusion matrixes. An overview of the classification results for the archetypal experiences and the explicit emotions is presented in Table 10. The first column of this table indicates the class labels used in the classification; other columns report the recognition accuracy.

**Table 7:** Confusion matrix of kNN classifier on the explicit emotions data set.

Classified as →	A-P	A-U	P-P	P-U
A-P	18	4	3	0
A-U	5	15	3	2
P-P	8	11	6	0
P-U	10	11	1	3

A-P: Active-Pleasant; A-U: Active-Unpleasant; P-P: Passive-Pleasant; P-U: Passive-Unpleasant.

**Table 8:** Confusion matrix of Naïve Bayes classifier on the explicit emotions data set.

Classified as →	A-P	A-U	P-P	P-U
A-P	16	4	2	3
A-U	4	13	5	3
P-P	6	7	6	6
P-U	6	7	7	5

A-P: Active-Pleasant; A-U: Active-Unpleasant; P-P: Passive-Pleasant; P-U: Passive-Unpleasant.

**Table 9:** Confusion matrix of LDA classifier on the explicit emotions data set.

Classified as →	A-P	A-U	P-P	P-U
A-P	11	4	3	7
A-U	5	9	4	7
P-P	4	5	15	1
P-U	5	5	3	12

A-P: Active-Pleasant; A-U: Active-Unpleasant; P-P: Passive-Pleasant; P-U: Passive-Unpleasant.

**Table 10:** Classification results obtained for the archetypal experiences and the explicit emotions.

Archetypes / Explicit emotions	kNN	Naïve Bayes	LDA
Anima, Animus, Hero, Shadow	46%	55%	53%
A-P, A-U, P-P, P-U	42%	40%	47%

A-P: Active-Pleasant; A-U: Active-Unpleasant; P-P: Passive-Pleasant; P-U: Passive-Unpleasant.

We also performed statistical comparison of the obtained classification accuracies with the chance level. For this purpose, it was necessary to break down the original data set into several groups. The power of the statistical test would be greater if the number of groups was larger. From this point of view, it would be desirable to have prediction models trained individually for each participant because this would result in the greatest number of groups. Unfortunately, this was not possible because each of the subjects viewed only one film clip of each category, and therefore, there were no data for training individual prediction models. For this reason, it was decided to split the data set into several groups that contained three participants each. According to combinatorics, there are 2300 ways to divide 25 participants into groups of three and we resampled our data set into 2300 groups.

Then, we built prediction models for each of these groups. Since quantity of the samples contained in each of the groups was significantly limited, the prediction models were created using kNN algorithm. This classification method can work reasonable well with small samples of data [66]. We also tried using two other algorithms introduced in the manuscript but they demonstrated classification accuracies below the chance level that clearly indicated inadequate amount of training data. Furthermore, leave-one-out cross-validation could not be used when training prediction models on small samples of data because the models were very sensitive to outliers and the validation fold could include an outlier. Therefore, we decided to set the number of folds for cross-validation equal to three.

Classification performance of the prediction models corresponding to the archetypes and the explicit emotions is presented in Table 11. As it can be seen from the table, accuracy of prediction models built for the small groups of participants was worse than accuracy of the model obtained for the original data set. This observation can be explained by small amount of training data (only 12 samples) available for the classification algorithm in each group of participants.

**Table 11:** Descriptive statistics for the classification results obtained with kNN method after resampling the original data set into groups of three participants.

Group	Archetypes	Explicit Emotions
Average	40.56%	39.64%
Std. Deviation	0.09	0.09
Std. Error	0.01	0.01

The prediction models were trained on the physiological data.

Having obtained classification results for 2300 groups of participants, paired-samples t-tests against chance level (25 percent) were performed for the archetypes and the explicit emotions. These statistical tests showed that for both types of the stimuli classification accuracies were significantly above the chance level [t(2299) = 75.038, p < 0.001 for archetypes and t(2299) = 73.010, p <. 001 for explicit emotions].

Moreover, we compared the results that are related to the archetypes and the explicit emotions using this data set. For this purpose, a paired-samples t-test was run where the classification accuracy corresponding to the film clips depicting different stimuli was treated as a dependent variable. The outcome of the test indicated that the film clips related the archetypal experiences were classified with higher accuracy than the ones related to the explicit emotions [t(2299) = 3.215, p = 0.001].

After analysis of the physiological data, we looked at the SAM ratings provided by the subjects after viewing the film clips. Their reports included information about three factors that are often used in emotion modeling: valence, arousal, and dominance. According to the dimensional theory of affect, the majority of a person’s affect variability is covered by a combination of these factors [67]. Similarly to analysis of the physiological data, we had to train several prediction models to answer the question of how accurately the archetypal experiences and the explicit emotions could be predicted based on the participants’ reports. The LDA method was used for this purpose. Analysis of the data corresponding to the archetypal clips indicated that a prediction model could achieve a classification accuracy of 33 percent. It was considerable above the chance level but could not outperform the results obtained using the physiological data. The same analysis was repeated for the explicit emotions. With the LDA classification method we could predict the explicit emotions based on the reports of the subjects with an accuracy of 51 percent. A comparison of these findings and the classification results obtained using the same method from the physiological data is presented in Table 12.

**Table 12:** Comparison of the classification performance for the physiological data and the introspective reports.

Archetypes / Explicit emotions	Physiological data	Introspective reports
Anima, Animus, Hero, Shadow	53%	33%
A-P, A-U, P-P, P-U	47%	51%

A-P: Active-Pleasant; A-U: Active-Unpleasant; P-P: Passive-Pleasant; P-U: Passive-Unpleasant.

In order to test whether the physiological signals provide significantly more accurate classification results of the archetypal experiences than the introspective reports, it was necessary to again resample the original data set into 2300 groups of three participants. Classification performance of the prediction models corresponding to the physiological signals and the introspective reports is presented in Table 13. Having obtained the classification results, a paired-samples t-test was performed for comparing accuracy levels between the physiological data and the introspective reports. The statistical test demonstrated that prediction models trained on the physiological data had a significantly higher accuracy than the ones obtained from the self-reports [t(2299) = 7.174, p < 0.001].

**Table 13:** Descriptive statistics for the classification results of the archetypal experiences obtained with kNN method after resampling the original data set into groups of three participants.

Group	Physiological Data	Introspective Reports
Average	40.56%	38.45%
Std. Deviation	0.09	0.09
Std. Error	0.01	0.01

The prediction models were trained on the physiological data and the introspective reports.

Furthermore, we statistically tested which data enabled us to train the most accurate prediction models for classifying the explicit emotions. Classification performance of prediction models for the explicit emotions is presented in Table 14. A paired-samples t-test was run for comparing accuracy of the models built using the physiological data and the introspective reports. It indicated that models based on the self-reports provided significantly higher number of correct predictions [t(2299) = 20.720, p < 0.001].

**Table 14:** Descriptive statistics for the classification results of the explicit emotions obtained with kNN method after resampling the original data set into groups of three participants.

Group	Physiological Data	Introspective Reports
Average	39.64%	46.28%
Std. Deviation	0.09	0.11
Std. Error	0.01	0.01

The prediction models were trained on the physiological data and the introspective reports.

## Discussion

According to Jung, people share certain impersonal traits, which do not develop individually but are inherited and universal. He introduced the concept of archetypes in order to describe the contents of the unconscious psyche. While it is not clear whether Jungian model is valid, the notion of archetypes found applications in various areas of psychological science. In this study, a comparison of two approaches for identification of archetypes in human experience was carried out: introspective reports and physiological measures.

### Physiological Measures

A number of statistical tests were run on the collected data. Their outcomes gave evidence of a significant relationship between some of the physiological signals and the psychological conditions of the subjects. Furthermore, the results of our experiment demonstrated that prediction models constructed with established data mining techniques and trained on the physiological data of the subjects achieved average classification accuracy. The models were obtained with three different classification methods (kNN, naïve Bayes, and LDA) featuring classification rates from 46 percent to 55 percent for the archetypal experiences. Explicit emotions could be recognized with an accuracy ranging from 40 percent to 47 percent. It is difficult to compare the results related to the archetypal experiences with the state of the art because we are aware of only two studies that examined them from the psychophysiological perspective. An experiment reported by Ivonin, et al. [68] could be considered as an exception but it only documented physiological reactions to one archetype. Therefore, the comparison is problematic. Another study by Ivonin, et al. [69] reported higher accuracies of predicting archetypal experiences but it used within-subjects models for classification, and moreover, fell short in conducting statistical comparison of classification accuracies obtained for different conditions. In order to have a relative benchmark, the results obtained for the explicit emotions can be set against previous studies that dealt with recognition of affect. Based on the review provided in [57], the predictive power of our models is on par with affect recognition studies in terms of classification accuracy. There are studies where higher accuracies have been reported, for instance in [70–72] researchers were able to achieve classification precision of up to 97.4 percent. While we acknowledge their accomplishments, it is necessary to take into account two types of limitations that seem to exist in these studies. First, the classification may be subject-dependent, meaning that recognition algorithms are trained and optimized to perform well with physiological data from a particular person. Second, the number of psychological states, which are predicted, is generally smaller than five. In fact, the greatest accuracy was obtained for the classification of only three affective states. However, the more classes need to be predicted, the more difficult the classification problem becomes. For example, in the case of two classes, accuracy of 50 percent is attained simply by chance, while in a situation with four classes the chance level is 25 percent.

Prior to the experiment, one of our concerns was that in both film clips and real life other factors may strongly influence emotional arousal and valence. Moreover, these effects may be sufficient to complicate recognition of the archetypal experiences with competing signals, i.e., we expected there to be a significant potential for confusion of the recognizer when confronted with variable emotional states. It also bears emphasis that emotion-influencing stimuli are likely more prevalent in day-to-day experience than archetype-inducing stimuli.

Based on our observations, one could conclude that this concern was not justified. It seems that each of the archetypes triggered a recognizable pattern of affective and cognitive reactions in the participants. These reactions led to activations in autonomic nervous systems of the subjects that were captured with the physiological sensors. Naturally, a superposition of the affective and cognitive responses forming an archetypal experience and other affective states could occur. Moreover, it is reasonable to assume that such overlays had place while the subjects were watching the film clips during this experiment, and from our point of view, they could not be avoided. This is likely one of the reasons why the classifier could not achieve accuracy higher than 55 percent. An important observation is that although the recognizers had to deal with competing signals, the performance was still significantly better than a chance level. This finding was confirmed by the statistical test executed on the original data set resampled into groups of three participants.

Overall, the experimental findings suggest a positive relationship between physiological responses of the subjects and the induced archetypal experiences. It was confirmed with a number of statistical tests, and moreover, we were able to train prediction models that differentiated between four archetypes with an accuracy of up to 55 percent. Prior to the study, we expected that classification performance would not be very high due to the complex and multidimensional nature of archetypal experiences. An interesting finding is that higher prediction accuracy was obtained for the archetypal data set comparing to the explicit emotions data set. One may propose a hypothesis that the archetypes were classified more accurately than the explicit emotions because, by definition, they elicit cognitive and affective activations that tend to be universal across the population. On the other hand, the explicit emotions are more subject-dependent and considerably vary due to personality differences. Moreover, this observation was statistically confirmed with a paired-samples t-test on 2300 subsets obtained from the original pool of physiological data.

Another finding related to the archetypal experiences was that classification models trained based on the physiological signals demonstrated significantly better accuracy than the models built on the introspective reports. A possible explanation for this observation could be that the subjects were not fully aware of unconscious emotions elicited with the archetypal film clips, and therefore, did not report them correctly via the self-reports. This hypothesis is supported by the fact that prediction models obtained from the introspective reports performed significantly more precise classification of the explicit emotions than the ones trained using the physiological data.

### Introspective Reports

Besides recording physiological responses of the subjects, after every film clip we asked them to provide reports about their feelings by means of the SAM ratings. As it turns out from our analysis (see Table 10), in case of the archetypal data set, the classifiers trained on the SAM data demonstrated poorer accuracy in comparison to the classifiers built based on the physiological recordings. An opposite observation was discovered with regard to the explicit emotions data set: the classifiers trained on the SAM data performed considerably better that the ones built using the physiological data. Further analysis of our results reveals that the subjects were capable to consciously differentiate the explicit emotions substantially better than the archetypal experiences. In the framework of Jung this finding seems reasonable. Indeed, if the participants were unaware of the archetypal nature of the demonstrated stimuli, how would they be able to consciously report about it? It appears that in these settings their unconscious minds responded to the presentation of the film clips and led to psychophysiological reactions of particular patterns. Another explanation for this finding is that the SAM tool may be better suited for capturing information about explicit emotions rather than complexes of emotions related to archetypes. Still, the SAM ratings seem to be one of the best available instruments for collection of the subjects’ feedback in the settings of our study because we are not aware of any tools designed specifically for archetypal experiences. While existing techniques for uncovering unconscious meaning (e.g., implicit association tests [73] or the forced metaphor elicitation technique [74]) may facilitate different experimental findings, they would have to be adjusted according to the design of this experiment and be focused on archetypes. These adjustments represent a research question on its own, and for this reason, we preferred to use only well-established SAM technique in our study.

### Archetypal Stimuli

An important aspect of this study was the selection of stimuli that were capable of inducing archetypal experiences. Film clips were found to be the most suitable media type for this purpose. Our method for the selection and confirmation of the validity of the archetypal stimuli took into account three principal aspects.

First, according to Rottenberg et al. [39], validation of film clips on the basis of self-reported emotional ratings is a significantly limited approach because even the most robust self-reported norms provide no guarantee that a film will elicit the desired emotional experience. In case of the films with archetypal appearances, it was reasonable to expect even less benefit in the application of this approach.

For this reason, it was decided to approach the problem of selecting and validating the archetypal stimuli in a qualitative manner. We contacted one of the most competent research organizations that specialize in the archetypal symbolism: The Archive for Research in Archetypal Symbolism (ARAS) associated with The C.G. Jung Institute of San Francisco. The film clips were then evaluated by a group of four experts from this organization. A pool of the film clips obtained through this collaboration was used in the present study.

Second, we applied the principle of triangulation [75] in order to increase the probability that the film clips elicit the expected archetypal experience. Healey [76] illustrated that the triangulation of multiple sources of information leads to a better set of affective labels. In this study, we combined the qualitative recommendations obtained from ARAS with the quantitative physiological data. The qualitative information provided the first round of validation. Next, the classification performance of the prediction models trained on the physiological data corresponding to the archetypal stimuli contributed to the second round of validation.

Third, we used the approach known as ‘Direct and Indirect Measures’ [77] for the measurement of the participants’ conscious awareness about the archetypal stimuli. According to this approach, the subjects are consciously aware of the effects of the stimuli if the sensitivity of the direct measure is greater or equal to the sensitivity of the indirect measure. In our study, the self-reports were assumed to fulfill the role of the direct measure and the physiological responses were considered as the indirect measure. As the analysis of the data collected in the experiment suggests, the indirect measure seemed to perform better than the direct measure in case of the archetypal stimuli.

Overall, the problem of selection and validation of the archetypal stimuli is challenging. This study represents one of the first steps in this direction. We hope to address this problem with a greater detail in the future research.

### Limitations

The present study has some limitations. First, the number of the subjects recruited for this experiment was relatively low. It seriously impacted performance of the statistical tests and prevented us from making stronger claims and conclusions. Another limitation is related to the small number of film clips used in the experiment. It would be beneficial to expand the pools of videos representing each of the archetypes and explicit emotions. Moreover, the generalizability of the experimental findings is limited. We did our utmost to ensure that the obtained models provided valid predictions by applying an appropriate statistical technique. However, the reported results should be repeated in several other studies for the final confirmation of their generalizability. Finally, the conclusions made here are limited to the types of stimuli presented (four archetypes and four explicit emotions) and cannot be generalized to other archetypes or emotions. Further work should be done to address other archetypes and emotions using various types of stimuli (e.g., music clips instead of video clips).

## Conclusion

While implicit mental processes of people are starting to receive an increased attention of the scientific community, it is still unclear how the unconscious side of the psyche operates. Scholars have proposed various theoretical frameworks for description of the psyche. One of them was developed by Jung who introduced the notion of archetypes. According to Jungian model, archetypes constitute the content of the collective unconscious depicting prototypical categories of people, objects, and situations. Although the overall validity of Jungian model remains an open question, the notion of archetypes has been adopted in many areas of psychological science. Moreover, it has also been extended to the consumer domain. Since the state of the art still lacks an established method for objective evaluation of individuals’ experiences related to archetypes, we aimed at providing a comparison of two promising approaches for accomplishment of this task. In our study, we analyzed the accuracy of identifying archetypal experiences of individuals using introspective reports facilitated with the SAM technique and physiological sensors for measurement of cardiovascular activity, electrodermal activity, respiration and skin temperature. Four archetypes and four explicit emotions were included in the study and presented to the subjects by means of film clips. The subjects were asked to provide introspective reports about their psychological states after watching the clips. Data mining methods were applied to the physiological recordings in order to construct prediction models that were able to recognize the elicited archetypes with an accuracy of up to 55 percent. The explicit emotion could be recognized with an accuracy of up to 47 percent. Evaluation of prediction models trained on the SAM data demonstrated that the archetypes could be differentiated with an accuracy of 33 percent while the explicit emotions were correctly predicted in 51 percent of cases. Based on these experimental findings, one could have an impression that the subjects had better conscious awareness about the explicit emotions pictured in the clips than about the archetypes. Overall, it seems that physiological signals may offer more reliable information about archetypal experiences of the individuals than introspective reports and the collected data set enabled us to confirm this statement statistically. On the other hand, we could provide statistical evidence that physiological signals were able to predict archetypal experiences significantly more accurately than the explicit emotions.
